# What Patients Can Tell Us: Topic Analysis for Social Media on Breast Cancer

**DOI:** 10.2196/medinform.7779

**Published:** 2017-07-31

**Authors:** Mike Donald Tapi Nzali, Sandra Bringay, Christian Lavergne, Caroline Mollevi, Thomas Opitz

**Affiliations:** ^1^ Institut Montpelliérain Alexander Grothendieck (IMAG) Department of Mathematics Montpellier University Montpellier France; ^2^ Laboratoire d'Informatique, de Robotique et de Microélectronique de Montpellier (LIRMM) Department of Computer Science Montpellier University Montpellier France; ^3^ Paul Valery University Montpellier France; ^4^ Biometrics Unit Institut du Cancer Montpellier (ICM) Montpellier France; ^5^ BioSP Unit Institut National de la Recherche Agronomique (INRA) Avignon France

**Keywords:** breast cancer, text mining, social media, unsupervised learning

## Abstract

**Background:**

Social media dedicated to health are increasingly used by patients and health professionals. They are rich textual resources with content generated through free exchange between patients. We are proposing a method to tackle the problem of retrieving clinically relevant information from such social media in order to analyze the quality of life of patients with breast cancer.

**Objective:**

Our aim was to detect the different topics discussed by patients on social media and to relate them to functional and symptomatic dimensions assessed in the internationally standardized self-administered questionnaires used in cancer clinical trials (European Organization for Research and Treatment of Cancer [EORTC] Quality of Life Questionnaire Core 30 [QLQ-C30] and breast cancer module [QLQ-BR23]).

**Methods:**

First, we applied a classic text mining technique, latent Dirichlet allocation (LDA), to detect the different topics discussed on social media dealing with breast cancer. We applied the LDA model to 2 datasets composed of messages extracted from public Facebook groups and from a public health forum (cancerdusein.org, a French breast cancer forum) with relevant preprocessing. Second, we applied a customized Jaccard coefficient to automatically compute similarity distance between the topics detected with LDA and the questions in the self-administered questionnaires used to study quality of life.

**Results:**

Among the 23 topics present in the self-administered questionnaires, 22 matched with the topics discussed by patients on social media. Interestingly, these topics corresponded to 95% (22/23) of the forum and 86% (20/23) of the Facebook group topics. These figures underline that topics related to quality of life are an important concern for patients. However, 5 social media topics had no corresponding topic in the questionnaires, which do not cover all of the patients’ concerns. Of these 5 topics, 2 could potentially be used in the questionnaires, and these 2 topics corresponded to a total of 3.10% (523/16,868) of topics in the cancerdusein.org corpus and 4.30% (3014/70,092) of the Facebook corpus.

**Conclusions:**

We found a good correspondence between detected topics on social media and topics covered by the self-administered questionnaires, which substantiates the sound construction of such questionnaires. We detected new emerging topics from social media that can be used to complete current self-administered questionnaires. Moreover, we confirmed that social media mining is an important source of information for complementary analysis of quality of life.

## Introduction

Social media such as Facebook, Twitter, or Internet forums dedicated to health-related topics have evolved into easily accessible participatory tools for the exchange of knowledge, experience, and opinions through structured collections of text documents [1]. Online health forums are used by patients to exchange information [2]. Patients maintain their anonymity while discussing freely with other patients. Whereas communication with doctors and the medical staff in hospitals mainly revolve around technical issues of the disease and treatment, social media give patients access to more general exchanges of information, experiences, and mutual support among former and current patients [3]. Such forums can therefore be considered as a valuable resource for the study of health-related quality of life (QoL). As shown by some studies (eg, [4]), the anonymous environment of social media facilitates the unbiased expression of opinions and of feelings such as doubt or fear. Internet users have been shown to be primarily interested in specific information on health problems or diseases [5-7] and in adopting a healthier lifestyle and looking for alternative points of view [5]. Here we propose an approach to structure and evaluate clinically relevant information in narratives extracted from online health social media, with a focus on the QoL of patients with breast cancer.

While constant progress in medical science leads to new treatments and improved chances to prolong lives, such treatments can be difficult to undergo. QoL can be considered as an alternative clinical end point in this context, moving the focus away from quantity to quality [8-11]. QoL falls within the scope of patient-reported outcomes; that is, measures of perceived health [12,13]. These measures must therefore be reported by patients themselves. For instance, alternative treatments such as palliative treatment of terminal cancer may be less efficient from a traditional clinical stance but may still be preferable with respect to the patients’ QoL [14,15]. Moreover, health economists must take into account the expense of treatments with respect to their effective benefits, for instance measured by the improvement in QoL (see Hirth et al [16] and Cutler and McClellan [17] for a general discussion, and Hillner and Smith [18] for a cost-effectiveness study of chemotherapy in certain cases of breast cancer).

Since QoL is a multidimensional, subjective, and culture-dependent concept, its quantification is not as straightforward, as shown in the literature review of Garratt et al [19]. This concept includes at least physical, psychological, and social well-being, as well as symptoms related to illness and treatment. Today, QoL is assessed in cancer clinical trials by self-administered questionnaires developed by the European Organization for Research and Treatment of Cancer (EORTC). The EORTC Quality of Life Questionnaire Core 30 (QLQ-C30) [20] is a generic self-administered questionnaire often associated with disease-specific modules, such as the EORTC breast cancer module (QLQ-BR23). The EORTC QLQ-C30 contains 30 items and evaluates 15 dimensions of QoL: 5 functional scales, 1 QoL and global health status scale, and 8 symptomatic scales, as well as 1 scale measuring the financial difficulties associated with the disease. The EORTC QLQ-BR23 contains 23 questions. It is usually administered with the EORTC QLQ-C30 and is designed to measure QoL for breast cancer patients at various stages and with different treatment modalities. The evaluation consists of 4 functional scales and 4 symptomatic scales. Usually, self-administered questionnaires evaluate functional and symptomatic dimensions and are filled in at a predefined time of the study protocol, such as at baseline, during treatment, and at follow-up. In this context, an advantage of social media is that they allow patients to leave a written trace of their sentiment at any time, therefore avoiding potential self-reporting bias owing to a change of perception due to time lag.

Opitz et al [21] developed an automated approach for the supervised detection of topics defined in QLQ-BR23 questionnaire items for cancerdusein.org, a French forum specialized in breast cancer. In this new work, we used an unsupervised method to discover topics covered by health social media. Unsupervised methods have been successfully applied to biomedical data. For example, Arnold and Speier [22] presented a topic model tailored to the clinical reporting environment that allows for individual patient timelines. Lu et al [23] used text clustering algorithms on social media data to discover health-related topics. Zhang et al [24] applied a convolutional neural network classifier to an online breast cancer community and carried out a longitudinal analysis to show topic distributions and topic changes throughout the members’ participation. In our study, the main medical application was to help improve questionnaires by including new topics of interest for patients (topics frequently discussed by patients and the impact on QoL) as new items in the questionnaires.

Researchers have developed several topic models, including latent semantic analysis [25], probabilistic latent semantic analysis [26], latent Dirichlet allocation (LDA) [27], and latent semantic indexing [28]. In this study, we defined a general process based on LDA [27] and applied this model to social media. LDA, an unsupervised generative probabilistic method for modeling a corpus, is the most commonly used topic modeling method. The main disadvantage of LDA is that there are no objective metrics that justify the choice of the hyperparameters. However, the main advantage of LDA is that it is a probabilistic model with interpretable topics. Nowadays, a growing number of probabilistic models are based on LDA and dedicated to particular tasks. For example, Zhan et al [29] used LDA to identify topics among posts generated by e-cigarette users in social media. Wang et al [30] and Paul and Dredze [31] constructed a specialized and advanced LDA model using biomedical terms to provide a more effective way of exploring the biomedical literature. LDA has also been successfully used for patient-generated data [32-36] and in particular for online breast cancer discussions [3,24]. Hao and Zhang [37] used LDA to examine what Chinese patients said about their physicians in 4 major specialty areas. Hao et al [38] used LDA to identify topics in positive and negative textual reviews of obstetricians and gynecologists from the 2 most popular online doctor rating websites in the United States and China. Yesha and Gangopadhyay [39] described methods to identify topics and patterns within patient-generated data related to suicide and depression. LDA has also been used as a feature to build machine learning models to automatically identify the extent to which messages contain emotional and informational support on online health forums dealing with breast cancer [40] or on Chinese social media [41].

Conducting automated research as we have done here is of considerable interest for processing a large amount of text obtained from social media. The LDA approach for extracting topics allows for better targeting for information exploration, reducing search time, and treating topics as a flat set of probability distribution; it can also be used to recover a set of topics from a corpus. In this work, we only used the LDA model and tuned parameters to align the topics found with QoL questionnaires. The originality of our approach is to automatically relate the topics obtained with the LDA method to the questionnaire items with an adaptation of the Jaccard coefficient.

In this study, the purpose of our approach was diverse: (1) to provide a nonconventional analysis of QoL from social media and put the topics identified with this nonconventional analysis into perspective with those of classical QoL questionnaires collected in clinical trials (in particular in breast cancer: EORTC QLQ-C30 and QLQ-BR23); (2) to apply the LDA model to patient data with relevant pretreatments; (3) to index the narratives with respect to topics extracted through an unsupervised statistical analysis of forum content and to predefined topics from questionnaires used in cancer clinical trials; and (4) to discover new topics directly from patients’ concerns that are not included in the current questionnaires used to evaluated QoL, with the possibility that these topics could be included in these questionnaires if sufficiently relevant.

## Methods

### Data

#### Data Description

In this work, we used datasets from 2 different social media sources: cancerdusein.org and Facebook groups. Table 1 summarizes statistics from these 2 datasets.

**Table 1:** Number of users, threads, and posts on a social network and a health forum analyzed in this study.

Characteristics	Health forum (cancerdusein.org)	Social network (Facebook groups)
Date	October 2010-October 2014	October 2010-October 2014
No. of users	675	1394
No. of discussion threads	1050	11,013
No. of messages	16,868	70,092

The first dataset contained the forum posts from cancerdusein.org, a French health forum with more than 16,000 posts. These posts cover a large number of topics related to health issues. This forum is recommended to patients in a brochure of the *Institut National du Cancer* (INCA), which is the French reference organization in oncology. The forum is recommended for patients to exchange information and find comfort and potential solutions to their problems. It serves as an online cancer support community, where cancer patients, cancer survivors, and their families share information about cancer and their conditions. The second dataset contains posts from groups on Facebook, one of the most well-known social networks. We extracted 70,092 posts from 4 different public groups or communities on Facebook: *Cancer du sein*, *Octobre rose 2014*, *Cancer du sein - breast cancer*, and *brustkrebs*. We collected data from groups focusing on the adult population (the targeted users) and in which users were very active.

On both social media platforms, patients freely exchange information without the need for moderators to supervise discussions. New messages can either be added to an existing thread or be posted to open a new thread. In cancerdusein.org, a thread appears in exactly 1 of the 13 predefined subforums, for example, *Discussion générale* [general discussion], *Vivre mon cancer au quotidien* [daily life with my cancer], *Les bonnes nouvelles* [good news], or *Récidives et combats au long cours* [relapses and long-term battles]. In Facebook groups, there are no predefined topics to index the threads. Structuring topics according to the subforum structure is possible in cancerdusein.org, but this structure underlines the relatively uninformative and widely spread topics, covering a strongly unbalanced number of messages. Such indexing is not possible in Facebook groups. Interestingly, we propose to accomplish a finer analysis of topics in the next section, which further enables the presence of several topics within 1 message.

#### Data Preprocessing

Texts on social media are often strongly heterogeneous and noisy, with many deviations from standards of spelling, syntax, and abbreviations, which impede efficient natural language processing. The French language has a rich spelling and grammar, characterized by special characters such as *ç*, various kinds of accented vowels (eg, *é*, *è*, *ê*, *ë*, *â*, and *à*), and many flexional variants. Additional rules exist for linking subsequent terms in certain situations (eg, the contraction *du* formed from *de+le* and the contraction *des* formed from *de+les)*. As a consequence, automatic correction of text not obeying those rules is relatively difficult in practice. Furthermore, semantic analysis of texts is complicated by a large number of homonymy relationships: for example, *pas* can either mean *step* (noun) or can be the negation adverb *not*. As Balahur [42] and Farzindar and Inkpen [43] have pointed out, these linguistic peculiarities may affect classification performance. For this reason, we developed the following preprocessing steps.

- *Removal of user tags.* All user tags that have been identified in our corpus are removed, for example, @name, @surname.
- *Replacement of hyperlinks and email addresses.* All the hypertext links are replaced by the term “link” and all the email addresses are replaced by the term “mail.” Hyperlinks (Internet, email, etc) are deleted. Emoticons are coded as :smile:, :sad:, etc.
- *Replacement of slang.* Some expressions frequently used on social media, such as lol, mdr[lol], and xD, are removed.
- *Lemmatization.* All words are lemmatized (using TreeTagger [44]).
- *Lowercasing.* Capitals letters are lowercased.
- Removal of stopwords.
- *Replacement of specific patient terms.* The texts for the 2 corpora are usually highly focused on a specific domain (breast cancer, in our case). Most often, as patients are laypersons in the medical field, they use slang, abbreviations, and their own vocabulary during their exchanges. To automatically analyze text from social networks, we need a specific vocabulary. In this work, we use the vocabulary created by Tapi Nzali et al [45] to replace the patients’ terms with biomedical terms used by health professionals and presented in shared medical resources. For example, *crabe* [crab] is replaced by *cancer*, *onco* is replaced by *oncologue* [oncologist].
- *Correction of spelling.* Spelling correction is important to remove redundant dimensions of data and to improve part-of-speech tagging, which is the basis for many statistical and rule-based methods in natural language processing. We apply spelling correction based on specialized dictionaries constructed ad hoc and the open source tool GNU Aspell version 0.60.6.1, whose algorithm proposes a list of possible corrections for unknown terms from the corpus. We use the following ad hoc dictionaries: lists of breast cancer drugs and of secondary effects, and proper names extracted from forum metadata (usernames, user residence) and from narratives (terms with capital first letter not at the beginning of a sentence; usernames identified from salutations at the beginning of forum posts).
- *Extraction and deletion of forum pseudonyms.* All the pseudonyms, previously extracted from each website, are used. The pseudonyms are extracted and deleted if they exist in the post.

### Unsupervised Topic Detection and Assigning

#### Modeling Topics With Latent Dirichlet Allocation

Today, detection of latent semantic structures and topics has become a very active field of research in the text mining community. We focused on the LDA model [27], which has become a standard model for unsupervised topic detection from a text corpus. It is a probabilistic model with a hierarchical definition of its components. With the LDA model, we generated new documents from a given model. Based on the relatively simple and robust bag-of-words representation of text documents, it leaves the order of occurrence of terms and sentence structure out for consideration. For a given corpus of *D* documents, we first defined the relevant vocabulary *V*, a preprocessed collection of terms occurring in the corpus. Typical preprocessing steps include spelling correction, lemmatization, and the removal of noisy or irrelevant terms. To define a topic *t*, we associated a nonnegative weight ω_ti_ with each of the vocabulary’s terms, *w*_i_, so that weights summed up to 1 (∑^V^_i=1_ ω_ti_=1). In practice, each topic typically consisted of a relatively small number of terms with nonnegligible weight. An LDA model uses a fixed number *K*>1 of topics. For each document *d*, weights ω_dt_≥0 indicate the occurrence probability of terms from topic *t*, where the sum of ω_dt_ over all topics *t* yields 1 (∑^K^_t=1_ ω_dt_=1). If document *d* contains *l*_d_ terms (or “positions”), we associated a topic *t*_dj_ with each of the positions *j*=1,..., *l*_d_, where the probability of associating topic *t* is α_dt_. Finally, each position was filled with a term, w_dj_, from the vocabulary, where the probability of using term *w*_i_ is ω_tdj_.

**Figure 1:**
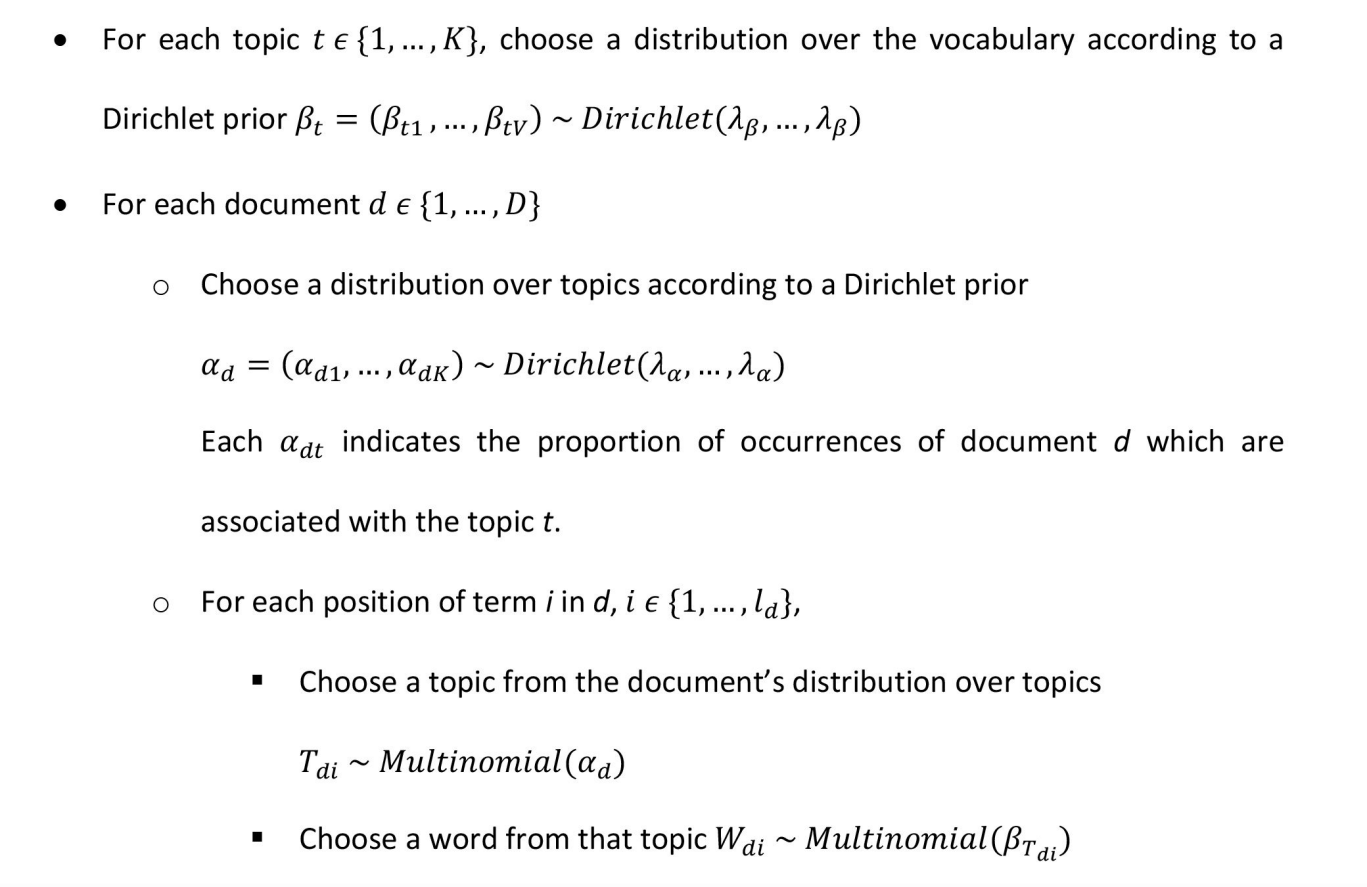
Algorithm proposing the corpus-generation model.

The corpus-generation model is proposed by the algorithm shown in Figure 1.

The principal information that we can learn from using such a model on a corpus of text data is the structure of represented topics and the distribution of topics over the documents contained in the corpus. The high number of unknown parameters in this model makes inference challenging, yet Bayesian techniques such as Gibbs sampling [46] have proven reliable. Based on prior assumptions about the distribution of the weights of terms in topics and of topics in documents on a range from very uniform to very spiked, these inference techniques are applied to the data to estimate the posterior distributions of the model. Most importantly, the most likely topic structure and the occurrence probabilities for topics in each document are proposed. In this work, we considered a message as a document.

#### Crucial Model Parameters

Besides *K*, 2 parameters often denoted as α and β strongly influence the distribution of topic probabilities for each of the messages. They are concentration parameters for the prior distributions of topics over a message (α) and of words over a topic (β). When α or β is smaller than 1 and decreases, prior mass concentrates closer and closer to the border of the simplex with spikes at each of its vertices. Then, 1 or fewer components (topics for α, words for β) carry strong probability in the mixture distribution. In the limit 0, a single component is selected with a probability of 1. On the contrary, when α or β is larger than 1 and increases, mass concentrates more and more in the barycenter of the simplex, leading to a mixture of the distribution, which is more and more balanced over all components. In the limit ∞, each component is selected with a probability of 1 over the number of components.

Now we will explain our choice of α based on the influence of α on the distribution of topic probabilities for messages and of term distributions for topics. When α=1, the prior distribution for the vector of topic probabilities corresponds to a uniform distribution on the simplex with *K* vertices. As α increases, the distribution concentrates more and more strongly toward the center of the simplex, such that most of the probabilities are closer to 1/ *K*. As α decreases, it concentrates more and more strongly toward the vertices, leading to some probabilities being further away from 1/ *K*. For fixed α, probabilities concentrate more and more around 1/ *K* as *K* increases. In Griffiths and Steyvers [47], values α=α_0_/ *K* with the constant α_0_=50 are encouraged, where dividing through *K* constantly keeps a certain complexity measure of the model. Exploratory analysis showed that α_0_=50 led to very flat probability vectors in our case, which made it difficult to attribute a small number of topics for indexation to each message. On the other hand, smaller values of α_0_ led to topics becoming more difficult to interpret due to flatter distribution of term probabilities within topics and similar dominating terms in multiple topics. After careful analysis of topics and posterior distributions for a range of values of α_0_, we decided to fix α_0_=10. Whereas higher values of α_0_ yielded a better fit of the model in terms of its likelihood, it led to very flat posterior probabilities for the topic distribution of messages. As in Griffiths and Steyvers [47], we decided to fix the value of parameter β to 0.1 for our experiments.

There is evidence [48] that automatic choice of parameters through a model selection criterion may result in an unsatisfactory topic collection, whose interpretation is more challenging than topics associated with suboptimal values of the criterion. Often, the calculation of held-out likelihood is used, allowing for approaches such as likelihood cross-validation. However, the likelihood calculation is not trivial, and some standard methods produce inaccurate results (see [49]).

#### Vocabulary Definition

To avoid noisy topics that are difficult to interpret, it is useful to focus on terms with potential medical relevance. Here, we defined terms as sequences of words, and often there was only a single word. To begin, we used terms indexed in the French version of the Medical Subject Headings (MeSH) [50]. Then we added terms figuring in a list of breast cancer drugs (extracted from the online resource) or appearing in a list of nonconventional treatments (extracted from the French Wikipedia entry). We denoted this term set as *MED*. We retained 481,111 occurrences of 18,672 terms in 16,868 messages on cancerdusein.org, and 626,043 occurrences of 18,741 terms in 70,092 messages on Facebook. The resulting topics, often strongly dominated by a single term, appeared to be rather difficult to interpret by clinical experts, possibly due to the relatively small dimension of the term-document space. We categorized terms figuring in the representative terms according to their grammatical role: nouns/proper names (*NN*), verbs (*V*), and adjectives (*A*). Then, we extracted topics by applying LDA to the original *MED* term set, extended by terms according to scenarios *MED+NN+V+A*. Based on the exploratory inspection of topics extracted by LDA in the approaches presented in the following, we further removed a small number of strongly represented terms leading to strong noise (*femme* [woman], *temps* [time or weather]), and medically meaningless topics.

### Align Topics and Questionnaires

**Figure 2:**
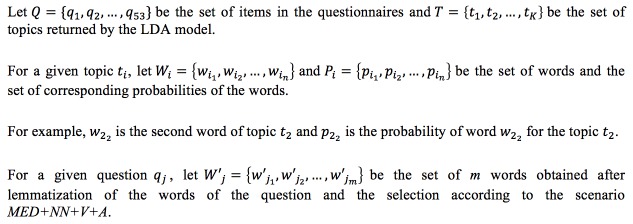
Automatic identification of correspondences between topics and questionnaires. LDA: latent Dirichlet allocation; *MED* + *NN* + *V* + *A*: set of medically relevant terms (*MED*) extended by terms categorized by their grammatical role (*NN*: nouns and proper names; *V*: verbs; *A*: adjectives).

**Figure 3:**
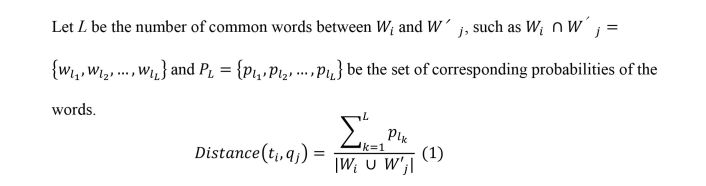
Equation to calculate the distance between a latent Dirichlet allocation topic and an item of the questionnaire.

With the topics returned by the LDA model, we automatically identified correspondences between the topics and the questionnaires, as shown in Figure 2. To align topics and questionnaires, we computed a distance between each question *q*_j_ and all topics *t*_i_ in *T.* We kept the topic with the higher distance. To compute the distance between an LDA topic and an item of the questionnaire, we customized the Jaccard coefficient [51] by taking into account the probability of the words obtained with the LDA model, as shown in Figure 3 (equation 1).

## Results

### Topic Modeling Result

To run experiments, we used the R package LDA [52] and the R environment version 3.2.5 (R Foundation) for the implementation. We tested different scenarios, and an expert validated and labeled the topics and verified the association between topics and questionnaires items. The expert is a biostatistician and QoL researcher in the cancer field [53,54].

In scenario *MED* + *NN*, most of the topics were of a factual nature, whereas scenario *MED* + *NN* + *V* led to a more complete description of topics, where verbs often add information about actions undertaken by users and other stakeholders (wait, consult, seek, support, etc) and about user sentiment (feel, cry, tire, fear, accept, etc). In scenario *MED* + *NN* + *V* + *A*, several topics consisting mainly of emotional words were difficult to interpret from a medical point of view. We reported the stability of the majority of topics that were identified through the scenarios *MED* + *NN*, *MED* + *NN* + *V*, and *MED* + *NN* + *V* + *A* due to the similarity of dominating terms. After careful analysis, we narrowed down the choice of *K* to a value between 20 and 30. With more than 20 topics, we found duplication of topics (2 topics may deal with the same subject). In addition, some are unable to be interpreted (the medical expert found no meaning). Consequently, we decided to retain scenario *MED* + *NN* + *V* + *A* with 20 topics. Finally, we fixed *K*=20 for the duration of this study. For each topic, we showed only 20 keywords having higher probabilities under that topic. These keywords were presented to the expert. Table 2 and Table 3 list the topic modeling results of the 2 corpora. We show the top 10 keywords for each topic. Table 4 shows the results of the 20 topics interpreted by the medical expert on the 2 corpora.

### Relationships Between Questionnaire Topics

In this work, we used 2 QoL questionnaires (EORTC QLQ-C30 and EORTC QLQ-BR23) to look for relationships between the studied dimensions in these previous questionnaires and topics that we interpreted. The EORTC QLQ-C30 is a 30-item, self-administered, cancer-specific questionnaire designed to measure QoL in the cancer population. The assessment comprises 5 functional scales (physical, role, cognitive, emotional, and social), 8 symptomatic scales (fatigue, nausea and vomiting, pain, dyspnea, insomnia, loss of appetite, constipation, and diarrhea), and 1 scale measuring financial difficulties and 1 measuring global health status and QoL by a score ranging from 0 to 100 through the 30 items [20]. The EORTC QLQ-BR23 is a 23-item, self-administered, breast cancer-specific questionnaire, usually administered with the EORTC QLQ-C30, designed to measure QoL in the breast cancer population at various stages and with patients with differing treatment modalities. The assessment comprises 4 functional scales (body image, sexual functioning, sexual enjoyment, and future perspective) and 4 symptomatic scales (systemic therapy side effects, breast symptoms, arm symptoms, and hair loss) [55]. The EORTC health-related QoL questionnaires are built on a Likert scale with polytomous items.

To find the theme corresponding to a question, we used equation 1 (Figure 3) proposed above. We obtained the following relationships:

- Topic *sexuality* is related to items 44 (To what extent were you interested in sex?) and 45 (To what extent were you sexually active?).
- Topic *hair loss* is related to item 34 (Have you lost any hair?).
- Topic *body care and body image during cancer* is related to items 39 (Have you felt physically less attractive as a result of your disease or treatment?) and 40 (Have you been feeling less feminine as a result of your disease or treatment?).

These relationships were validated by a medical expert. Following validation of the results, we calculated the precision. On cancerdusein.org data, for the 53 items, 39 relationships with topics were validated by the medical expert and 14 were invalidated, for a precision of 74%. On Facebook data, for the 53 items, 36 relationships were validated by the medical expert and 17 were invalidated, for a precision of 68%. The medical expert also manually examined the invalidated relationships. This step reduced the time spent by the expert to find relationships between the questions and the topics. The obtained precision rates can be explained by the fact that the items of the questionnaires are composed of very short sentences. On average, these sentences contain fewer than 5 words.

**Table 2:** Top 10 frequently occurring words for the first 10 topics (among the 20 found) on cancerdusein.org forum data.

Topic no.	Top 10 words with their translation		Topic label^a^
	French	English translation	
1	*cheveu*, *perdre*, *perruque*, *tomber*, *tête*, *commencer*, *repousser*, *chimiothérapie*, *perte*, *foulard*	hair, lose, wig, fall, head, begin, regrowth, chemotherapy, loss, scarf	Hair loss
2	*prendre*, *temps*, *travail*, *demander*, *soin*, *reprendre*, *charge*, *travailler*, *aide*, *payer*	take, time, job, ask, care, restart, charge, work, help, pay	Work life during cancer and financial aspects
3	*effet*, *chimiothérapie*, *secondaire*, *cure*, *douleur*, *passer*, *mammographie*, *nausée*, *docétaxel*, *fatigue*	effect, chemotherapy, secondary, treatment, pain, pass, mammography, nausea, docetaxel, fatigue	Chemotherapy and its secondary effects
4	*prendre*, *effet*, *douleur*, *traitement*, *problème*, *tamoxifène*, *prise*, *penser*, *secondaire*, *arrêter*	take, effect, pain, treatment, problem, tamoxifen, catch, think, secondary, stop	Hormone therapy and its secondary effects
5	*sein*, *bras*, *chirurgie*, *reconstruction*, *opération*, *douleur*, *prothèse*, *opérer*, *enlever*, *cicatrice*	breast, arm, surgery, reconstruction, operation, pain, prosthesis, operate, remove, scar	Breast reconstruction
6	*baiser*, *petit*, *beau*, *super*, *attendre*, *soutien*, *nouveau*, *guerrier*, *grand*, *vérité*	kiss, little, beautiful, great, wait, support, new, warrior, big, truth	Support from patient’s family and friends
7	*ongle*, *peau*, *radiothérapie*, *main*, *séance*, *pied*, *rayon*, *brûlure*, *crème*, *conseil*	nail, skin, radiotherapy, hand, session, foot, radius, burn, cream, council	Radiotherapy and its secondary effects
8	*prendre*, *manger*, *boire*, *essayer*, *miel*, *aider*, *produit*, *demander*, *santé*, *complément*	take, eat, drink, try, honey, help, product, ask, health, complement	Complementary and alternative medicine
9	*lire*, *forum*, *message*, *venir*, *nouveau*, *donner*, *trouver*, *site*, *réponse*, *écrire*	read, forum, message, come, new, give, find, site, response, write	Media and forum information exchange
10	*homonymie*, *enfant*, *fille*, *maman*, *vie*, cancer, *vérité*, *vivre*, *malade*, *famille*	homonymy, child, girl, mom, life, cancer, truth, live, sick, family	Family background and breast cancer

^a^Topic label was assigned by a medical expert.

**Table 3:** Top 10 frequently occurring words for the first 10 topics (among the 20 topics found) on Facebook data.

Topic no.	Top 10 words		Topic label^a^
	French	English translation	
1	*voir*, *attendre*, *résultat*, *médecin*, *oncologie*, *examen*, *biopsie*, *mammographie*, *contrôle*, *scanner*	see, wait, result, doctor, oncology, examination, biopsy, mammography, test, scanner	Diagnosis
2	*douleur*, *effet*, *chimiothérapie*, *secondaire*, *jour*, *prendre*, *mal*, *fatigue*, *nausée*, *chaleur*	pain, effect, chemotherapy, secondary, day, take, bad, fatigue, nausea, heat	Chemotherapy and its secondary effects
3	*justice*, *moral*, *garder*, *aller*, *fort*, *dureté*, *battre*, *étape*, *force*, *combat*	justice, morale, keep, go, strong, hardness, beat, step, strength, fight	Breast cancer as a daily battle
4	*cheveu*, *perdre*, *tomber*, *repousser*, *perruque*, *couper*, *raser*, *tête*, *joli*, *foulard*	hair, lose, fall, growth, wig, cut, shave, head, beautiful, scarf	Hair loss
5	*prendre*, *suivre*, *dire*, *soin*, *arrêter*, *traitement*, *tamoxifène*, *poids*, *perdre*, *homonymie*	take, follow, tell, care, stop, treatment, tamoxifen, weight, lose, homonymy	Secondary effect of treatment
6	*aller*, *justice*, *passer*, *sexologie*, *allergologie*, *baiser*, *penser*, *meilleur*, *voir*, *reposer*	go, justice, pass, sexology, allergology, kiss, think, best, see, rest	Body care and body image during cancer
7	*homonymie*, *dire*, *vérité*, *suivre*, *peur*, *sexologie*, *comprendre*, *croire*, *dureté*, *enfant*	homonymy, tell, truth, follow, fear, sexology, understand, believe, hardness, child	Family background and breast cancer
8	*demander*, *suivre*, *droit*, *travail*, *aide*, *médecin*, *payer*, *charge*, *travailler*, *donner*	ask, follow, law, job, help, doctor, pay, charge, work, give	Work life during cancer and financial aspects
9	*sein*, *opération*, *reconstruction*, *enlever*, *bras*, *opérer*, *mastectomie*, *cicatrice*, *retirer*, *prothèse*	breast, operation, reconstruction, remove, arm, operate, mastectomy, scar, withdraw, prosthesis	Breast reconstruction
10	*suivre*, *aller*, *fille*, *sol*, *voir*, *rire*, *regarder*, *marier*, *croire*, *lire*	follow, go, girl, ground, see, laugh, look, marry, believe, read	Support from patient’s family and friends

^a^Topic label was assigned by a medical expert.

**Table 4:** List of identified topic titles with *K*=20 in collaboration with an expert.

Topic no.	cancerdusein.org	Facebook
1	Hair loss	Diagnosis
2	Work life during cancer and financial aspects	Chemotherapy and its secondary effects
3	Chemotherapy and its secondary effects	Breast cancer as a daily battle
4	Hormone therapy and its secondary effects	Hair loss
5	Breast reconstruction	Secondary effects of treatments
6	Support from patient’s family and friends	Body care and body image during cancer
7	Radiotherapy and its secondary effects	Family background and breast cancer
8	Complementary and alternative medicine	Work life during cancer and financial aspects
9	Media and forum information exchange	Breast reconstruction
10	Family members with breast cancer	Support from patient’s family and friends
11	Treatment period	Interaction with nurses and doctors
12	Everyday life during cancer	Anxiety and fatigue
13	Healing	Healing of family member
14	Search for medical information	Relapse
15	Mourning	Sexuality
16	Diagnosis	Body care and body image during cancer
17	Breast cancer as a daily battle	Family members with breast cancer
18	Body care and body image during cancer and sexuality	Healing
19	Surgery	Support from patient’s family and friends
20	Waiting for results of analysis, concerns	Treatment period

Table 5 shows the relationships between topics from questionnaires and those we found in the 2 corpora. The first column lists the topics of the 2 questionnaires, with the corresponding questionnaires items shown in column 2. Columns 3 and 4 give the corresponding topics obtained with LDA in the 2 corpora. Table 6 shows the percentage of documents belonging to each topic in cancerdusein.org and Facebook. We noticed that the numbers of messages belonging to each topic are almost equal; this shows the importance of all the topics that we found and that were discussed by patients.

#### Data From cancerdusein.org

We succeeded in interpreting the 20 topics obtained from the output of our model on the cancerdusein.org corpus. Table 2 presents the 10 first topics and the top 10 words obtained by our model that were interpreted by an expert. Some relationships were established. In the QLQ-C30, we found matches for all of the topics except for global health status and QoL. In the QLQ-BR23 form, we matched all of the topics.

#### Data From Facebook

We succeeded in interpreting the 20 topics obtained from the output of our model on the Facebook corpus. Table 3 presents the 10 first topics and the top 10 words obtained by our model that were interpreted by an expert. Some relationships were established. In the QLQ-C30, we found matches for all of the topics except for role functioning, cognitive functioning, and global health status and QoL. In the QLQ-BR23 form, we matched all of the topics.

## Discussion

We have presented what we believe to be the first study of health social media data in French, as a potential source of analysis of the QoL for breast cancer patients. We used accurate machine learning models to identify topics discussed in online breast cancer support groups. Then we examined the relationships between the discovered topics and studied dimensions from QoL self-administered questionnaires. Exploratory and in-depth analysis of these data is a potential source of candid information as an alternative to analysis of QoL based on self-administered questionnaires.

### Limitations

#### Patient-Authored Text

The first limitation of this study is the type of users, which produced the patient-authored text exploited in our process. Indeed, unless a group has formal gatekeeping of members, it is difficult to know for sure whether people posting to a forum or in a Facebook group are patients, survivors, health care professionals, care providers, family, or friends of patients. Consequently, topics extracted with our method may have been generated by users who do not have breast cancer. In particular, it has been known for decades that health information is sought principally by friends or family members, and then after that by patients [56]. In this work, we assumed that the relatives’ topics of interest were similar to patients’ topics of interest. However, in a previous work [57], we proposed a method to automatically deduce the role of the forum user. This method can be used at the beginning of our chain to exclude the posts of individuals who are not actual patients.

#### Generalization of the Method

The second limitation is that we harvested data from only 1 forum and different Facebook groups. However, this forum is frequently recommended by French physicians to patients. It is also recommended by INCA, which is the French reference organization in oncology. We deliberately selected this forum and these Facebook groups to examine similarities and differences within and between these 2 particular communities. Of course, there are certainly many other online communities related to breast cancer, and the users in these 2 online communities were not necessarily representative of users of all breast cancer social media.

It is also important to note that our method can be easily applied to other diseases. For example, we can (1) use brain cancer forum data to align topics discussed by patients with items of the EORTC QLQ-C30 and the brain cancer module (QLQ-BN20) [58] questionnaires, and (2) use lung cancer forum data to align topics discussed by patients with items of the QLQ-C30 and the lung cancer module (QLQ-LC13) [59] We have already also applied a similar approach to study other social media data such as Twitter [60]. The main adaptation is relative to the acquisition of the patient terms, which are specific to the disease and the social media as mentioned in the Data Preprocessing section above.

#### Latent Dirichlet Allocation Model

A third limitation was the choice of LDA. LDA requires much manual tuning of its parameters, which vary from task to task. We spent a lot of time finding the best parameters so that the results could be interpreted meaningfully. Such analysis makes itself a sort of “overfitting” to the task at hand, making it very hard to generalize the method to other datasets and other tasks. However, we efficiently defined parameters of 2 types of text (forum and Facebook posts), which can be reused for other studies on comparable corpora.

Topics covered on social media focused on a specific domain, breast cancer. It was difficult to adjust the number of topics because topics were closed: all of the users were discussing breast cancer. When we adjusted the model and sought the optimal *K* with methods such as those used in other studies (eg, [47,61,62]), we obtained more than 50 topics. An interesting perspective was using the heuristic approach defined by Zhao et al [63] to determine an appropriate number of topics. This method is based on the rate of perplexity change [62,64]. This measure is commonly used in information theory to evaluate how well a statistical model describes a dataset, with lower perplexity denoting a better probabilistic model [63]. Finally, as in Arnold et al [65], we observed that an expert is not able to interpret so many topics. In this study, we manually fixed *K*=20. We interpreted all the topics with minimal redundancies.

**Table 5:** Distribution of documents on each topic on cancerdusein.org and Facebook.

Questionnaires and their scales			Questionnaire items	cancerdusein.org	Facebook
**EORTC QLQ-C30^a^**					
	**Functional scales**				
		Physical functioning	1-5	Everyday life during cancer	Treatment period
				Treatment period	
		Role functioning	6, 7	Everyday life during cancer	
		Emotional functioning	21-24	Diagnosis	Diagnosis
				Breast cancer as a daily battle	Breast cancer as a daily battle
				Waiting for results of analysis, concerns	Anxiety and fatigue
				Support from patient’s family and friends	Support from patient’s family and friends
		Cognitive functioning	20, 25	Search for medical information	
				Media and forum information exchange	
		Social functioning	26, 27	Support from patient’s family and friends	Support from patient’s family and friends
				Work life during cancer and financial aspects	Work life during cancer and financial aspects
	**Symptom scales**				
		Fatigue	10, 12, 18	Chemotherapy and its secondary effects	Anxiety and fatigue
					Secondary effects of treatments
		Nausea and vomiting	14, 15	Chemotherapy and its secondary effects	Secondary effects of treatments
		Pain	9, 19	Chemotherapy and its secondary effects	Secondary effects of treatments
				Surgery	
		Dyspnea	8	Chemotherapy and its secondary effects	Secondary effects of treatments
		Insomnia	11	Chemotherapy and its secondary effects	Secondary effects of treatments
		Appetite loss	13	Chemotherapy and its secondary effects	Secondary effects of treatments
		Constipation	16	Chemotherapy and its secondary effects	Secondary effects of treatments
		Diarrhea	17	Chemotherapy and its secondary effects	Secondary effects of treatments
		Financial difficulties	28	Work life during cancer and financial aspects	Work life during cancer and financial aspects
**Global health status**					
	Global health status and quality of life		29, 30		
**EORTC QLQ-BR23^b^**					
	**Functional scales**				
		Body image	39-42	Breast reconstruction	Breast reconstruction
				Body care and body image during cancer, and sexuality	Body care and body image during cancer
				Surgery	
		Sexual functioning	44, 45	Body care and body image during cancer, and sexuality	Sexuality
		Sexual enjoyment	46	Body care and body image during cancer, and sexuality	Sexuality
		Future perspectives	43	Healing	Healing
					Relapse
	**Symptom scales**				
		Systemic therapy	31-34	Chemotherapy and its secondary effects	Secondary effects of treatments
		Side effects	36-38	Hormone therapy and its secondary effects	Chemotherapy and its secondary effects
		Breast symptoms	50-53	Breast reconstruction	Breast reconstruction
				Radiotherapy and its secondary effects	
				Surgery	
		Arm symptoms	47-49	Breast reconstruction	Breast reconstruction
				Surgery	
		Hair loss	35	Hair loss	Hair loss
**Topics without a relationship**					
				Complementary and alternative medicine	
				Mourning	
					Family background and breast cancer
				Family members with breast cancer	Family members with breast cancer
					Healing of family member

^a^EORTC QLQ-C30: European Organization for Research and Treatment of Cancer Quality of Life Questionnaire Core 30.

^b^QLQ-BR23: breast cancer module.

**Table 6:** Distribution of documents in each topic on cancerdusein.org and Facebook.

Topic no.	cancerdusein.org (n=16,868) n (%)	Facebook (n=70,092) n (%)
1	978 (5.80)	3294 (4.70)
2	590 (3.50)	3925 (5.60)
3	1147 (6.80)	3785 (5.40)
4	860 (5.10)	4065 (5.80)
5	1315 (7.80)	2804 (4.00)
6	759 (4.50)	3715 (5.30)
7	810 (4.80)	3014 (4.30)
8	523 (3.10)	3084 (4.40)
9	877 (5.20)	3645 (5.20)
10	692 (4.10)	3505 (5.00)
11	675 (4.00)	2804 (4.00)
12	523 (3.10)	2734 (3.90)
13	1113 (6.60)	5047 (7.20)
14	692 (4.10)	3014 (4.30)
15	843 (5.00)	2804 (4.00)
16	1063 (6.30)	2734 (3.90)
17	1248 (7.40)	3575 (5.10)
18	540 (3.20)	5607 (8.00)
19	1198 (7.10)	3432 (4.90)
20	422 (2.50)	3505 (5.00)

### Relationships Between Self-Administered Questionnaires and Social Media

We were able to match most of the topics from QoL self-administered questionnaires in social media. These topics correspond to a total of 95% (22/23) of topics in the cancerdusein.org corpus and 86% (20/23) of topics in the Facebook corpus. These figures underline the importance of studying QoL, because they correspond to patients’ real concerns. The topics that corresponded with those of the EORTC QLQ-C30 and the EORTC QLQ-BR23 questionnaires were hair loss, work life during cancer and financial aspects, chemotherapy and its secondary effects, breast reconstruction, support from the patient’s family and friends, treatment period, healing, diagnosis, breast cancer as a daily battle, body care and body image during cancer and sexuality, hormone therapy and its secondary effects, radiotherapy and its secondary effects, media and forum information exchange, everyday life during cancer, search for medical information, surgery, waiting for results of analysis, concerns, secondary effects of treatments, interaction with nurses and doctors, anxiety and fatigue, and relapse.

### Emerging Topics in Social Media

We also found 5 topics that are not present in QoL questionnaires. These topics correspond to a total of 15% (3/20) of the cancerdusein.org corpus and 15% (3/20) of the Facebook corpus. Of the 5 topics that do not appear in the questionnaires, 2 focus on patients. The emerging topics are complementary and alternative medicine, mourning, family background and breast cancer, family members with breast cancer, and healing of a family member. Among these 5 topics, we believe that 2 of them (complementary and alternative medicine, and family background and breast cancer) could be added to the QoL questionnaires. The topic complementary and alternative medicine focuses on nonconventional treatments and corresponded to a total of 3.10% (523/16,868) of the cancerdusein.org corpus. The topic family background and breast cancer focuses on the relationships of patients with their family, especially healing and grieving for a family member. This topic corresponded to a total of 4.30% (3014/70,092) of the Facebook corpus. The 3 others topics are not related to QoL. These topics deal with mourning, having family members with breast cancer, and healing of a family member. They were discussed by relatives of patients and not by patients.

### Different Uses of Forums and Social Networks

One of the reasons that led us to use 2 data resources (social networks and a health forum) was to discover the topics discussed in each platform. Table 7 presents the relationships between topics found in both social media and the percentage distribution of messages in each topic. Of 20 topics detected by our model in the corpus forum and Facebook, we found 11 common topics in the 2 corpora. Some of them have a similar frequency of discussion (Table 6). These topics are hair loss, work life during cancer, support from patient’s family and friends, treatment period, diagnosis, and family members with breast cancer. We observed that topics such as chemotherapy and its secondary effects, breast reconstruction, and breast cancer as daily battle were discussed more on the forum than on Facebook, maybe because the subject is more technical. As Table 7 shows, we noted that the topics support from a patient’s family and friends, body care and body image during cancer, and sexuality were discussed more on Facebook than on the forum because of visibility to friends. In the end, the topics discovered were quite similar. However, we observed a difference of length in the posts. Most of the time, posts from the health forum were longer than posts from Facebook. Even if the topics found in both social media were similar, messages from the forum provided more information and were better interpreted than messages from Facebook.

**Table 7:** Relationships between topics found on both social media (cancerdusein.org and Facebook) with *K*=20 in collaboration with an expert.

Topic names		cancerdusein.org (n=16,868)		Facebook (n=70,092)		Matched to questionnaire item
		Topic no.	n (%)	Topic no.	n (%)	
**Topics on both social media**						
	Hair loss	1	978 (5.80)	4	4065 (5.80)	Yes
	Work life during cancer and financial aspects	2	590 (3.50)	8	3084 (4.40)	Yes
	Chemotherapy and its secondary effects	3	1147 (6.80)	2	3925 (5.60)	Yes
	Breast reconstruction	5	1315 (7.80)	9	3645 (5.20)	Yes
	Support from patient’s family and friends	6	759 (4.50)	10	3505 (5.00)	Yes
				19	3432 (4.90)	Yes
	Family members with breast cancer	10	692 (4.10)	17	3575 (5.10)	No
	Treatment period	11	675 (4.00)	20	3505 (5.00)	Yes
	Healing	13	1113 (6.60)	18	5607 (8.00)	Yes
	Diagnosis	16	1063 (6.30)	1	3294 (4.70)	Yes
	Breast cancer as a daily battle	17	1248 (7.40)	3	3785 (5.40)	Yes
	Body care and body image during cancer, and sexuality	18	540 (3.20)	6	3715 (5.30)	Yes
				15	2804 (4.00)	Yes
				16	2734 (3.90)	Yes
**Topics on only 1 social media**						
	Hormone therapy and its secondary effects	4	860 (5.10)	N/A^a^	N/A	Yes
	Radiotherapy and its secondary effects	7	810 (4.80)	N/A	N/A	Yes
	Complementary and alternative medicine	8	523 (3.10)	N/A	N/A	No
	Media and forum information exchange	9	877 (5.20)	N/A	N/A	Yes
	Everyday life during cancer	12	523 (3.10)	N/A	N/A	Yes
	Search for medical information	14	692 (4.10)	N/A	N/A	Yes
	Mourning	15	843 (5.00)	N/A	N/A	No
	Surgery	19	1198 (7.10)	N/A	N/A	Yes
	Waiting for results of analysis, concerns	20	422 (2.50)	N/A	N/A	Yes
	Secondary effects of treatments	N/A	N/A	5	2804 (4.00)	Yes
	Family background and breast cancer	N/A	N/A	7	3014 (4.30)	No
	Interaction with nurses and doctors	N/A	N/A	11	2804 (4.00)	Yes
	Anxiety and fatigue	N/A	N/A	12	2734 (3.90)	Yes
	Healing of member family	N/A	N/A	13	5047 (7.20)	No
	Relapse	N/A	N/A	14	3014 (4.30)	Yes

^a^N/A: not applicable.

### Conclusions

In this work, we used an unsupervised learning model known as LDA to detect the different topics on a health forum and social network discussed by patients. We demonstrated how we used the LDA model on patient data with relevant preprocessing applied to 2 datasets obtained from a forum and Facebook messages. We used MeSH as the principal resource for medical terms and for patients’ and doctors’ vocabulary [45]. We automatically detected relationships between topics and questions. We found good relationships between detected topics and the dimensions of internationally standardized questionnaires used for breast cancer patients, which substantiate the sound construction of such questionnaires. We detected new emerging topics from social media that could be used to complete actual QoL questionnaires. Moreover, we confirmed that social media can be an important source of information for the study of QoL in the field of cancer.

In our ongoing work [21], we are targeting the classification of whole messages or text snippets with respect to the role of the narrator (patient, confidant of a patient, expert, health professional) and to the location within the trajectory of care (before or after an operation, first cancer or relapse). One potential limitation of this work was the number of topics (*K*=20) selected for our LDA model. This limitation may be overcome by using the number of topics for which the model is better adjusted [47,61,62], then, first, to merge topics that are close, and second, to find topics that could not be interpreted by humans and eliminate them. Moreover, the actual comparison of the 2 corpora (Facebook and forum) was done manually by the expert. A possibility is to adapt equation 1 (Figure 3) used to align LDA topics and questionnaire items in order to automatically compare topics extracted from the 2 corpora.

Of course, the lack of informed consent given by social media users for data usage leads to ethical questions. In particular, confidentiality with respect to the publication of research results is an issue (see others’ discussion and guidelines [66-68]). We adhered to those guidelines. We have presented results with a degree of detail that does not permit conclusions on individual users to be drawn. In the long term, we will study emotions described by patients in their messages for each topic and make some statistical analyses. Finally, we will use the emotion classification system built by Abdaoui et al [69] to detect polarity (positive, negative, or neutral), subjectivity (objective, subjective), and feelings (joy, surprise, anger, fear, etc) of users’ messages, and we will relate this information to the detected topics in order to determine patients’ perception of their disease. What are the topics that frighten patients the most and that need prevention?

## Abbreviations

EORTC: European Organization for Research and Treatment of Cancer
INCA: Institut National du Cancer
LDA: latent Dirichlet allocation
MeSH: Medical Subject Headings
QLQ-C30: Quality of Life Questionnaire Core 30
QoL: quality of life
